# Correction: Intact memory for local and distal cues in male and female rats that lack adult neurogenesis

**DOI:** 10.1371/journal.pone.0199905

**Published:** 2018-06-25

**Authors:** Desiree R. Seib, Erin Chahley, Oren Princz-Lebel, Jason Scott Snyder

There is an error in reference 25. The correct reference is: Yagi S, Chow C, Lieblich SE, Galea LA. Sex and strategy use matters for pattern separation, adult neurogenesis, and immediate early gene expression in the hippocampus. Hippocampus. 2016;26: 87–101. pmid:26179150
